# DNA methylation changes in young female adolescents with anorexia nervosa : focus on puberty related genes

**DOI:** 10.1007/s40618-026-02830-6

**Published:** 2026-02-27

**Authors:** Stefania Palumbo, Domenico Palumbo, Grazia Cirillo, Francesca Aiello, Giuseppina Rosaria Umano, Maria Gloria Gleijeses, Emanuele Miraglia del Giudice, Marco Carotenuto, Filomena Salerno, Anna Grandone

**Affiliations:** 1 Department of Womens and Childrens Health and General and Specialized Surgery, University of Campania Luigi Vanvitelli, Naples, Italy; 2 Laboratory of Molecular Medicine and Genomics,Department of Medicine, Surgery and Dentistry SMS, University of Salerno, Salerno, Italy; 3 Clinic of Child and Adolescent Neuropsychiatry, Department of Mental Health, Physical and Preventive Medicine, University of Campania Luigi Vanvitelli, Naples, Italy; 4 Integrated Pediatric Care Department, AOU Luigi Vanvitelli University Hospital, Naples, Italy

**Keywords:** Anorexia, Epigenetics, Methylation, Amenorrhea, Puberty

## Abstract

**Purpose:**

Anorexia nervosa (AN) is a severe eating disorder with high morbidity that typically arises during adolescence. While genetic predisposition contributes to its development, emerging evidence highlights the role of environmental stressors and epigenetic mechanisms—particularly DNA methylation—in mediating these effects. This study aimed to investigate genome-wide DNA methylation alterations in young female adolescents with AN and functional hypogonadotropic hypogonadism, compared to healthy pubertal controls, to explore in particular potential epigenetic biomarkers of metabolic dysfunction and pubertal delay.

**Methods:**

Peripheral blood DNA was extracted from 10 young adolescent girls with AN and 11 age-matched healthy controls. Genome-wide DNA methylation analysis was performed using the Infinium MethylationEPIC BeadChip (850 K) array, which interrogates over 850,000 CpG sites. Differentially methylated regions (DMRs) and CpG sites were identified, followed by functional annotation. Ingenuity Pathway Analysis (IPA) was conducted to investigate the biological significance of the methylation changes.

**Results:**

A total of 87 DMRs were identified, with 69% showing hypomethylation in the AN group. These included genes such as CLU and MKRN3, involved in hypothalamic regulation and pubertal timing. Additionally, 2072 differentially methylated CpG sites were found, affecting genes linked to metabolism (e.g., LEPR, NR1H3) and puberty (e.g., GHR, DLK1). Ninety-two genes encoding zinc-finger proteins, including MKRN3, showed altered methylation. IPA revealed enrichment in pathways related to leptin, sirtuin, estrogen, and GnRH signaling.

**Conclusion:**

Young female adolescents with AN exhibited distinct methylation profiles, primarily hypomethylation, affecting genes involved in energy homeostasis and pubertal development. These epigenetic alterations may represent biomarkers of metabolic dysfunction and pubertal delay in AN.

**Supplementary Information:**

The online version contains supplementary material available at 10.1007/s40618-026-02830-6.

## Background

Anorexia nervosa (AN) is a severe eating disorder characterized by an obsessive desire to lose weight and a disrupted body image, leading to extreme caloric restriction and severe malnutrition, which, in the most severe cases, can be fatal [1]. This condition is often associated with high rates of morbidity and comorbidities, including anxiety, depression, and other mental disorders [2]. Although AN affects individuals of all genders and age, most existing studies have focused on adult populations or older adolescents, with an average age well beyond the completion of puberty [3–5].

However, in recent years, there has been an increase in cases of AN in paediatric populations—occurring before or during early adolescence—with clinical features that differ from those observed in later-onset cases [6, 7]. This trend raises specific questions regarding how AN interferes with ongoing growth and pubertal maturation and whether epigenetic changes differ in this younger age group compared to adults. Puberty, in fact, represents a particularly vulnerable window for the development of eating disorders in females. Hormonal changes, including fluctuations in estrogen and gonadotropins, together with body image revaluations accompanying sexual maturation, increase the risk of onset and chronicity of AN during early adolescence [8–10]. Conversely, AN is known to disrupt normal pubertal development: affected girls frequently present with primary or secondary amenorrhea as consequence of severe caloric restriction and marked weight loss [9, 11]. These conditions lead to a functional reduction in gonadotropin secretion (LH and FSH), resulting in a state of functional hypogonadotropic hypogonadism. Such endocrine alterations are not mere comorbidities but reflect profound metabolic imbalances that may have long-term consequences for bone health, fertility, and overall wellbeing [12].

In parallel, over the past decade, epigenetics—particularly DNA methylation—has emerged as a crucial link between genetic predisposition and environmental influences, such as stress, dietary restriction, and metabolic alterations [13].

More recently, genome-wide DNA methylation studies (EWAS) have provided a more detailed and specific insight into epigenetic alterations in patients with anorexia nervosa. Several EWAS have identified differential methylation in genes involved in energy metabolism, insulin and leptin signalling, immune and inflammatory pathways, as well as neuronal and stress-related processes [3, 14]. Among these, the series of studies conducted by Steiger and colleagues represents the most comprehensive epigenomic investigations in AN to date. Using both cross-sectional and longitudinal designs, Steiger et al. demonstrated that DNA methylation patterns differ between patients in the acute phase of the disease, individuals recovering from it, patients in long-term remission, and healthy controls [21, 22] showing that many methylation changes are dynamic and partially reversible with nutritional rehabilitation, indicating that a substantial proportion of epigenetic alterations in AN are dependent on clinical status rather than representing a trait marker of the disorder.

Despite the robustness of these EWAS results, existing studies have almost exclusively examined cohorts of adults or older adolescents, often including patients with long disease duration, pharmacological treatments, or completed pubertal development. Consequently, it is unclear whether similar epigenetic signatures are already present in very young adolescents with early-onset AN, particularly during active pubertal transition. Based on existing data in the literature on anorexia nervosa, we decided to extend the analysis of DNA methylation profiles to a small cohort of girls with an average age of approximately 13 years suffering from AN, hypothesising that we would observe methylation changes overlapping with those reported in older cohorts, particularly in pathways related to energy metabolism, appetite regulation, immune response, and neuroendocrine signaling [3, 14]. In addition, this study specifically explores epigenetic alterations in genes involved in pubertal timing and reproductive axis regulation, including members of the zinc finger family—areas not yet systematically investigated in previous EWAS on anorexia nervosa—and investigates the role of transcription factors associated to differentially methylated genes involved in metabolic adaptation and pubertal dysfunction in early-onset anorexia nervosa. Given the key role of these genes in the onset of puberty, gonadotropin regulation, and energy status-dependent reproductive suppression, we hypothesise that their epigenetic modulation may represent an adaptive molecular mechanism linking metabolic stress to pubertal delay in early-onset AN. In this perspective, the study aims to identify possible early molecular correlates of the metabolic and endocrine alterations observed in paediatric AN.

## Results

### Population

Clinical and laboratory characteristics of the entire cohort are shown in Table 1. Subject with anorexia nervosa (AN) presented a similar mean age and pubertal stage than pubertal controls (CTP).

Healthy controls presented a slightly reduced height than AN patient but their auxological profile reflected constitutional variants rather than endocrine or nutritional pathology as the comprehensive clinical and endocrinological assessments excluded pathological growth retardation, confirming growth trajectories within familial/genetic targets and normal BMI SDS.

A statistically significant difference in BMI SDS existed between patients with ANs and controls (-1.71 ± 0.71 and 0.32 ± 1.03, respectively; p = < 0.001) as expected. Significantly lower levels of LH and 17-beta-estradiol were found in patients with ANs (*p*= .009 and 0.008, respectively). No difference in FSH levels between the two groups existed (Table 1).

In patients with AN, four subjects presented with primary amenorrhea while six patients had secondary amenorrhea with an initially normal onset of menarche (mean age at menarche 11.25 ± 0.69) and a mean amenorrhea period of 1.3 years (± 0.98 SDS).

**Table 1:** Clinical and laboratory characteristics of patients with AN

	AN	CTP	*p*-value
**Number**	10	11	
**Age years**	13.64 ± 1.91	13.88 ± 1.96	0.784
**BMI**,** SDS**	-1.71 ± 0.71	0.32 ± 1.03	< 0.001
**Height**,** SDS**	-0.28 ± 0.68	-1.44 ± 1.07	0.02
**Tanner Stage**,** breast**	2.5 ± 0.60	3 ± 0.76	0.184
**Tanner stage**,** pubic hair**	2.7 ± 0.42	3 ± 0.59	0.352
**LH**,** IU/L**	1.08 ± 1.06	5.2 ± 2.50	0.009
**FSH; IU/L**	6.9 ± 2.53	5.14 ± 2.48	0.403
**17β-estradiol**,** pg/mL**	16.68 ± 8.05	38.78 ± 9.43	0.008

The parameters are expressed as mean ± standard deviation, except for Tanner stage reported as median (min and max value). Mann-Whitney U Test Calculator and T-Test Calculator for 2 Independent Means were used as appropriate to calculate t-test and p-value for AN versus control group

## Psychological assessment

Among the instruments analyzed in this study for AN group, scores obtained on the Children’s Depression Inventory (CDI) indicated clinically significant depressive symptoms in the majority of participants (75%, 6 out of 8), with scores ranging from 19 to 48 (clinical cut-off: 19). Although depressive symptoms were detected on the Children’s Depression Inventory (CDI), clinical interviews ruled out the presence of DSM-IV [15] criteria for a diagnosis of major depressive disorder.

Similarly, the SCARED questionnaire revealed clinically relevant anxiety levels across multiple subscales, with scores reaching up to 65 (clinical cut-off: 25).

The CBCL results documented widespread difficulties. The mean total competence score exceeded the borderline threshold (M = 67.5; cut-off > 64), while scores for internalizing (M = 72.5) and externalizing problems (M = 65.3) frequently felt within the clinical range, indicating the presence of concurrent emotional and behavioral issues.

As summarized in Table 2, the distribution of scores across the primary assessment instruments revealed a clear predominance of clinically significant findings in multiple domains, including depressive symptoms, anxiety, and both internalizing and externalizing behavioral disturbances.

Overall, internal consistency was good to excellent for the psychometric instruments mentioned used with Cronbach’s alpha values in all case > 0.7 (i.e. 0.96 (CDI), 0.92 (SCARED-B), 0.90 (SCARED-G), EAT 0.93 and 0.90 (CBCL) respectively).

**Table 2:** Clinical features in patients with AN

**Age of onset (years)**	12.42 ± 1.91
**Cumulative illness duration (months)**	14.5 ± 17.43
**Child Behavior Checklist (CBCL)**,** total**	68.33 ± 2.51
**CBCL**,** comp.pro**	66.9 ± 6.52; 66 (55–78)
**CBCL**,** dist.int**	71.1 ± 10.6; 73 (52–90)
**CBCL**,** dist.est**	61.7 ± 7.7; 61 (49–76)
**Eating Attitudes Test (EAT)**	36.2
**SCARED b scale**	36.5 ± 13.6; 31 (22–65)
**SCARED g scale**	35.4 ± 12.6; 35 (18–61)
**Child depression inventory (CDI)**	22.5 ± 15.8; 19 (6–48)
**Secondary amenorrhea (%)**	60%
**Age at menarche (years)**	11.25 ± 0.76
**Months of menses presence before secondary amenorrhea (months)**	32.4 ± 0.6
**Duration of amenorrhea (months)**	15.6 ± 1.1

## Differentially methylated genomic regions in AN compared to healthy pubertal girls

To assess global DNA methylation changes that occur in girls with AN and CTP, we first identified differentially methylated regions (DMRs). This analysis revealed 87 DMRs (with an FDR < 0.05 and |ΔB| > 5%), of which 68.97% were hypomethylated in girls with anorexia who presented primary or secondary amenorrhea. These 87 DMRs are mainly included in the promoter regions of genes involved in immune and inflammatory pathways, such as *LTA*, in the hypothalamic feeding regulatory pathways, such as *CLU*, as well in genes involved in the onset of puberty, such as *MKRN3* (Additional file 1). We also applied the epigenetic Landscape in Silico Deletion Analysis (LISA) to identify enriched transcription factors potentially regulating genes associated with our DMRs. Among the top twenty significant transcription factors (Additional file 2), we found the progesterone receptor (PR) and estrogen receptor (ESR1) genes. Additionally, we also identified DNMT1 (DNA Methyltransferase 1), SFMBT1 (Scm-like with Four MBT Domains 1), and DNMT3B (DNA Methyltransferase 3 Beta) which are involved in DNA methylation processes and chromatin remodeling affecting gene expression.

## Differences between groups at individual CpG sites

**Fig. 1:**
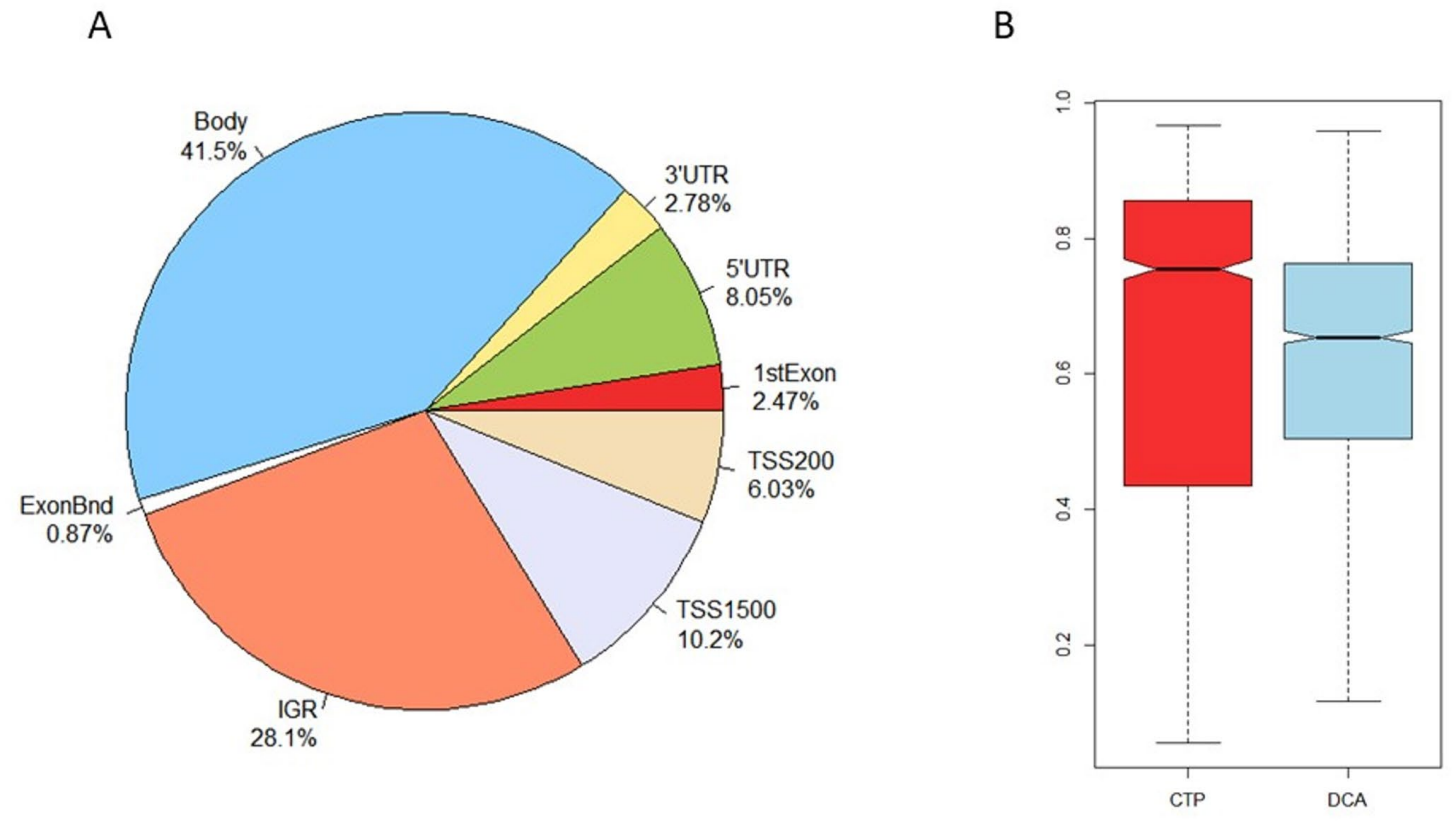
Distribution of the differentially methylated CpG sites identified for each comparison along the several genomic features**.A** The pie chart shows the percentage of DMPs for each genomic localization. On the right (**B**), the boxplots show the beta-values distribution per group (red for CTP, light blue for AN)

Since promoter methylation is known to influence gene regulation, we focused on CpG sites located at transcription start sites or within promoter regions. We identified 2072 differentially methylated probes (DMPs) mapping onto 1896 genes (Additional file 3). The comparison between girls with AN and CTP reported a significant proportion of differentially methylated CpGs (FDR < 0.05 and |ΔB| > 10%) located in intergenic regions (IGRs) and within the body of genes. Specifically, approximately 27% of these DMPs were located in promoter regions, including TSS1500 (10.2%), TSS200 (6.03%), 5’UTR (8.05%) and the first exon (2.47%) (Fig. 1A). Interestingly, the global analysis of DMPs showed the same trend as previously reported in DMRs, with lower methylation levels in the AN group compared to the controls (Fig. 1B). The comparison between the AN and control groups revealed several differentially methylated probes related to nutrition. In detail, genes involved in cholesterol and lipid processing (PRKAG2), glucose metabolism (IGF2R, RPTOR and SORBS1) and appetite regulation (GHRL, LEPR and CAMKK2). Other probes mapped onto genes relevant to immune function (such as NOD1, DSE, IRAK3, HHLA2, and NR1H3).

**Fig. 2:**
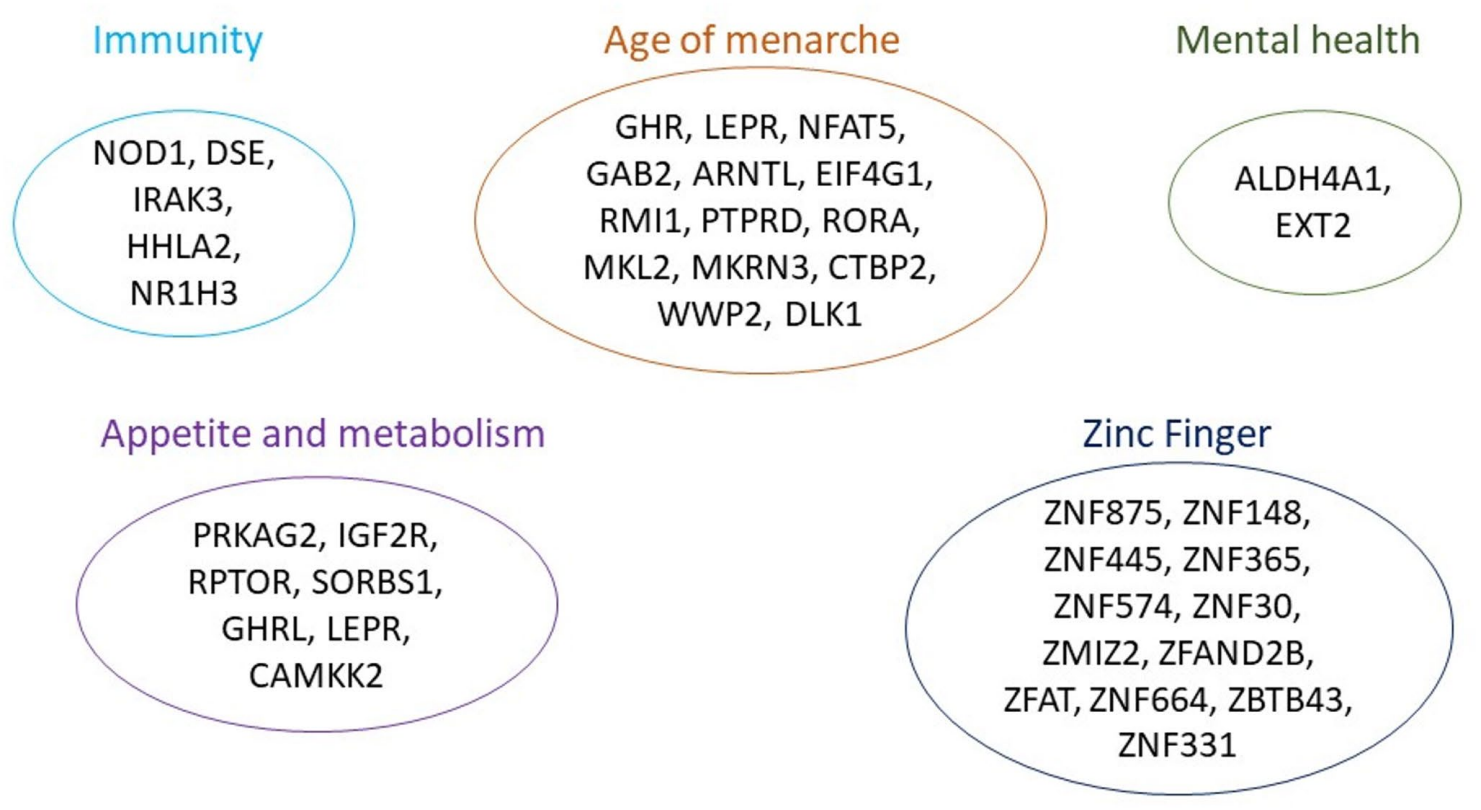
Differentially methylated genes. Genes with a different methylation between patients with AN and healthy pubertal controls divided by macro-areas of interest

Among hypermethylated CpGs sites, there were some located near genes involved in the regulation of anxiety and depression as serotonin degradation signaling (ALDH4A1 and EXT2) (Fig. 2). Additionally, we found a different methylation of CpGs sites in genes related to wound healing processes (TNXB) [3], skeletal homeostasis (LRP5), and energy metabolism (FHL2) [5].

## DMPs related to puberty

As shown in Table 3, fourteen DMPs mapped onto genes that have been previously associated with age of menarche: GHR, LEPR, NFAT5, GAB2, ARNTL, EIF4G1, RMI1, PTPRD, RORA, MKL2, MKRN3, CTBP2, WWP2, and DLK1 [3].

Transcription factors (TF) analysis detected in total 180 TFs (FDR < 0.05). The top 20 (full list shown in Additional file 2), include the receptor of sexual hormones as AR, ESR1, and PR that are crucial for reproductive processes such as menstrual cycle regulation and pregnancy. Additionally, the CEBPB factor which plays a role in the regulation of genes involved in immune response, metabolism, and differentiation of various cell types including adipocytes and hepatocytes, is also present in the list with a high score. Moreover, among genes with differentially methylated promoters there was an enrichment of genes containing Estrogen Responsive Elements (EREs) (hypergeometric test with p-value < 0.05), suggesting that some of the effects of estrogen signaling in puberty are modified through epigenetic mechanisms. However, we could not find a correlation between beta-values and estrogen levels, no CpGs were found directly correlated with them (data not shown).

### Ingenuity pathways analysis (IPA)

**Fig. 3:**
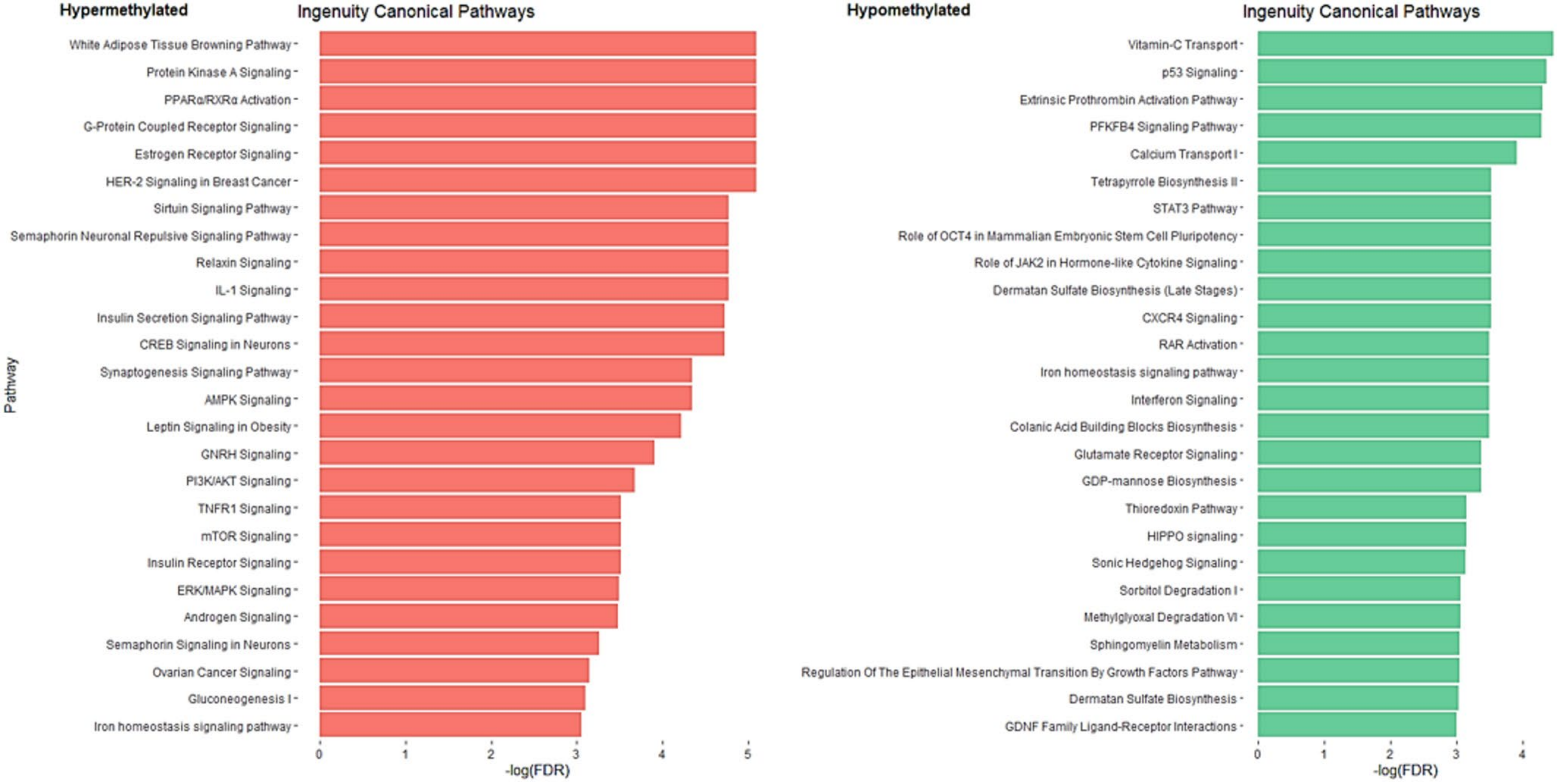
Ingenuity canonical pathway analysis between AN vs. CTP. In red are represented the pathways enriched with hypermethylated CpGs while, in green, the hypomethylated ones. The figure shows only part of the total pathways. The total list of pathways is available in additional file section

To identify pathways that could be modulated by the differentially methylated genes, we performed Ingenuity Pathway Analysis (IPA). Interestingly, our results showed differential methylation in signaling pathways that are easily correlated with reduced caloric intake, affecting metabolic processes and body mass. These include the white adipose tissue browning, sirtuin signaling, as well as insulin and leptin signaling. Additionally, IPA showed enrichment in neuronal signaling pathways such as the GnRH and semaphorin which have been previously reported to be associated with pubertal development [3, 5]. Other signaling pathways, as those involved in estrogen and ovarian cancer, present a different methylation status between the AN and control groups (Fig. 3). The entire list of differentially methylated pathways can be found in Additional file 4.

**Table 3:** Genes previously associated with the age of menarche [16, 17]

CG_ID	*P*.Value	adj.*P*.Val	deltaBeta	CHR	Gene name	Feature
**cg17942553**	**3.71E-06**	**0.000133**	**-0.10629**	**1**	**ARNT**	**TSS1500**
**cg15205441**	**2.51E-08**	**4.35E-06**	**-0.16401**	**10**	**CTBP2**	**5’UTR**
**cg02795603**	**7.87E-08**	**9.17E-06**	**-0.10834**	**3**	**EIF4G1**	**5’UTR**
**cg24252966**	**2.97E-07**	**2.29E-05**	**-0.12395**	**3**	**EIF4G1**	**5’UTR**
**cg04116507**	**1.07E-08**	**2.49E-06**	**-0.12154**	**11**	**GAB2**	**5’UTR**
**cg15466952**	**1.62E-07**	**1.51E-05**	**-0.10346**	**1**	**LEPR**	**5’UTR**
**cg02761778**	**1.87E-07**	**1.68E-05**	**-0.18258**	**16**	**NFAT5**	**5’UTR**
**cg25172788**	**8.39E-06**	**0.000238**	**-0.12531**	**9**	**RMI1**	**5’UTR**
**cg17181046**	**2.98E-07**	**2.30E-05**	**-0.20816**	**15**	**RORA**	**TSS1500**
**cg09789698**	**5.07E-09**	**1.53E-06**	**-0.1106**	**16**	**WWP2**	**5’UTR**
**cg16103098**	**0.002119**	**0.012164**	**0.172079**	**14**	**DLK1**	**TSS1500**
**cg00215587**	**0.000396**	**0.003707**	**-0.10076**	**15**	**MKRN3**	**5’UTR**
**cg24779990**	**0.000257**	**0.002724**	**-0.14077**	**5**	**GHR**	**TSS200**
**cg11196813**	**0.002353**	**0.013115**	**0.124879**	**9**	**PTPRD**	**TSS1500**
**cg20931294**	**1.58E-05**	**0.000372**	**-0.11603**	**16**	**MKL2**	**5’UTR**

## ZNF methylation

The methylation profiling analysis has identified 92 DMPs mapped near zinc finger (ZNF) genes. Some of these, such as ZNF148, which regulates the GABA transporter, and ZNF445 and ZNF365, which are involved in methylation processes and genomic stability, respectively, were previously reported by our group in another cohort of patients with pubertal disorders [16]. Additionally, other ZNF genes, such as ZNF574, ZNF30, ZMIZ2, and ZFAND2B, have been associated with the development of estrogen-dependent tumors, including breast and ovarian cancers. Notably, several differentially methylated ZNF genes are associated with metabolic syndrome, such as ZNF664, or with conditions like hypocholesterolemia and diabetes, such as ZNF860. Others, like ZFAT, are involved in adipocyte differentiation and body fat distribution. Furthermore, some genes, including ZBTB43 and ZNF331, are linked to changes induced by caloric restriction.

## Discussion

Anorexia nervosa during adolescence represents a particularly critical condition, as it coincides with key phases of growth and pubertal development. In this context, epigenetic mechanisms are increasingly recognized as potential mediators between environmental stressors, nutritional deprivation, and endocrine dysfunction. However, studies focusing on DNA methylation in younger patients with AN remain scarce, with most evidence deriving from adults or young adults.

Building on these considerations, our cross-sectional study specifically explored DNA methylation profiles in peripheral blood leukocytes from adolescent girls with AN and amenorrhea. We identified 87 differentially methylated regions and 2,072 CpG sites, with a higher percentage of reduced methylation in girls with AN. Based on previous EWAS studies in anorexia nervosa, particularly those by Steiger and Booij [3, 18, 19], we expected to observe widespread DNA methylation changes in genes involved in energy metabolism, immune response, and neuroendocrine regulation. The global hypomethylation pattern observed in our cohort is therefore consistent with, and represents a replication of, prior findings in a younger population. Nutritional deficiencies, including a lack of methyl donors such as folates [20, 21], due to prolonged fasting in AN may contribute to the hypomethylation observed in these patients. This potential link suggests that caloric restriction may lead to epigenetic modifications potentially affecting gene expression and hormonal balance [22, 23].

We observed epigenetic changes in genes central to metabolic, nutritional, and immune functions, echoing previous studies [3, 19]. In particular, Steiger et al. [19] conducted longitudinal studies which demonstrated that DNA methylation is dynamic and responsive to nutritional rehabilitation, with many alterations normalizing in remission. However, our design differed substantially (cross-sectional vs. longitudinal), and Steiger’s cohort was larger (*n* = 75 or 145) [18, 19] and included older individuals, some with pharmacological treatment. Methodologically, while Steiger used combined arrays (450 K and EPIC BeadChips), our study used only EPIC BeadChips, with broader CpG coverage. Despite these differences, we observed several overlapping findings. Both studies identified differential methylation in genes involved in glucose and insulin metabolism (e.g., RPTOR, SORBS1, IGF2R), appetite regulation (CAMKK2, GHRL), and energy homeostasis (PRKAG2) [24]. Additionally, we reported methylation changes in genes related to immune response (e.g., NOD1, IRAK3) and genes previously associated with AN as TNXB [25]. Notably, CpG sites within the NR1H3 gene were hypermethylated in patients with ANs, consistent with previous findings by Booij et al. [3]. NR1H3 is a regulator of macrophage activity and plays a key role in lipid metabolism and inflammation [26], with knockout mouse models further supporting its function in cholesterol homeostasis [27]. These functions suggest that NR1H3 may contribute to molecular mechanisms relevant to hunger, inflammation, and lipid balance in the pathophysiology of AN.

. While several methylation changes replicate previous findings in anorexia nervosa, others involve genes related to pubertal regulation and reproductive axis control. Given that amenorrhea and delayed puberty are hallmark features of anorexia nervosa in adolescent girls [28–30], these results point to epigenetic pathways beyond core anorexia-related genes that have not been systematically examined in prior EWAS and should therefore be considered exploratory. A particularly notable finding was the hypomethylation of the MKRN3 gene, a known inhibitor of gonadotropin-releasing hormone (GnRH) secretion and pubertal onset [31, 32]. Reduced methylation in MKRN3 may indicate a loss of epigenetic repression, potentially contributing to the hypothalamic hypogonadism observed in AN [33]. This observation is consistent with the idea that profound metabolic and endocrine disruptions due to caloric restriction underlie pubertal delay in AN. Given the lack of previous epigenome-wide studies focusing on puberty-related genes in anorexia nervosa, this observation should be regarded as hypothesis-generating rather than confirmatory. However, further studies are needed to determine whether MKRN3 methylation changes translate into altered expression and functional outcomes. We also detected differential methylation in genes related to pubertal timing and reproductive health, including GHR, LEPR, GAB2, and notably DLK1 [17]. DLK1 (Delta-Like 1 Homolog), part of the non-canonical Notch signaling pathway, is a key regulator of pubertal development, energy metabolism, and adipocyte differentiation [34–36]. It also modulates brown and white fat balance and bone development, systems commonly affected in AN. While DLK1 has not been directly associated with anorexia, its role in regulating energy homeostasis suggests a plausible indirect involvement in the disorder’s metabolic and developmental features. Additionally, our study revealed that differentially methylated CpGs were enriched in genes involved in estrogen and GnRH signaling, further supporting the notion that anorexia disrupts the delicate hormonal balance necessary for normal pubertal development [37]. These pathways are known to play a crucial role in reproductive health, and their epigenetic deregulation in AN could provide insights into the observed delay in menarche and ongoing reproductive dysfunction in affected individuals. In fact, the search for transcription factors targeting DMPs revealed higher scores for hormone receptors such as progesterone, androgens and estrogens, suggesting that epigenetics may modulate hormone action and response. As reported by Tremolizzo et al. [5], steroid hormone levels in patients with ANs were inversely correlated with overall DNA methylation in the blood. The correlation analysis between estrogen levels and DMPs in our cohort did not yield significant results, probably due to the cross-sectional nature of the study and the limited number of the samples. However, a significant number of DMPs were identified within or in close proximity to genes containing high-affinity estrogen-responsive elements that could be involved in pubertal timing and endocrine development, confirming the findings of previous longitudinal studies on normal pubertal transition [38, 39].

An interesting aspect of our findings is the enrichment of differential methylation in zinc finger proteins (ZNFs), a large family of transcription factors involved in chromatin remodeling, metabolism, pubertal timing, and imprinting [40]. In total, 92 ZNF genes showed altered methylation in patients with AN. While direct links between these ZNFs and eating disorders are currently lacking, several are involved in biological pathways potentially affected by chronic undernutrition or hormonal changes. For example, ZNF664 [41, 42] and ZFAT [43] are implicated in metabolic regulation and fat distribution; ZBTB43 and ZNF331 are associated with responses to caloric restriction [44, 45]; and ZNF148 regulates GABAergic neurotransmission and insulin signaling [46] as well ZNF445 and ZFP57 are known regulators of imprinting at loci such as MEG3/DLK1, with possible relevance to pubertal development [47–49]. Moreover, the presence of SNPs near ZNF genes linked to age at menarche in GWAS further suggests their involvement in reproductive maturation [40]. These gene-level observations are complemented by our pathway enrichment analysis, which highlighted signaling systems involved in hormonal regulation, energy metabolism, and pubertal development—such as estrogen, GnRH, sirtuins, leptin, insulin, and semaphorin pathways [50–52]. Furthermore, we observed differential methylation in the G protein-coupled receptor (GPCR) signaling pathway, which plays a key role in neurosensory and endocrine regulation, including insulin secretion, β-cell function, and glucose homeostasis [53]. GPCRs are also involved in neurotransmitter activity (e.g., serotonin, noradrenaline), potentially linking them to the psychiatric features of AN such as depression [54]. In addition, we identified altered methylation in leptin and insulin pathways—both crucial in metabolic regulation. Leptin and insulin levels are known to be markedly reduced in underweight patients with ANs and correlate with BMI and body fat [55]. Other energy-related pathways, such as AMP-activated protein kinase (AMPK) and adipose tissue browning, also showed methylation changes. Notably, Bredella et al. [56] found that brown adipose tissue (BAT) is associated with higher bone mineral density and lower Pref-1 (DLK1) levels, suggesting a possible protective role of BAT on bone health in AN.

Even though the reported data are interesting, we acknowledge several limitations of the present study, including the small sample size, the cross-sectional design, and the use of peripheral blood rather than disease-relevant tissues. Although blood may reflect systemic epigenetic patterns [57], future investigations should adopt longitudinal and multi-omics approaches (e.g., RNA-Seq, ChIP-Seq) and implement more refined patient stratification to reduce potential confounders. Another potential limitation is related to the control group: some patients were in fact referred for short stature, however in none of them was the diagnosis confirmed and in all of them organic causes were excluded. Despite these limitations, our findings provide preliminary evidence supporting the involvement of epigenetic alterations in anorexia nervosa, particularly in pathways related to metabolism and reproductive function. Importantly, the cross-sectional design prevents any causal inference. The observed methylation differences cannot establish whether these epigenetic marks precede, result from, or represent adaptive responses to the metabolic and endocrine alterations induced by chronic undernutrition. We hypothesize that the hypomethylation observed in genes regulating pubertal timing and reproductive function—such as *MKRN3*, *DLK1*, *LEPR*, and *GHR*—may reflect a reversible, energy-dependent mechanism aimed at temporarily downregulating the gonadotropic axis under caloric restriction. Within this framework, epigenetic modulation of puberty-related genes could act as an adaptive pathway linking metabolic stress to delayed pubertal progression, rather than as a primary etiological factor of the disorder.

Future longitudinal studies assessing methylation dynamics before and after nutritional rehabilitation will be essential to determine whether these epigenetic signatures normalize with weight restoration and recovery of gonadal function. The potential reversibility of these methylation changes highlights their possible clinical utility as biomarkers for disease monitoring or as targets for therapeutic intervention. Nevertheless, all interpretations should remain cautious, and our data should be regarded as exploratory and hypothesis-generating, forming a basis for future large-scale investigations.

Taken together, our findings extend existing epigenetic research in anorexia nervosa by combining the replication of previously reported disease-associated alterations with exploratory signals implicating pubertal and reproductive regulatory pathways in early-onset disease. This integrated pattern reflects a study design aimed not at a purely pilot investigation, but at expanding current epigenetic knowledge into a critical and previously understudied developmental window. Overall, these biologically plausible results complement the existing literature and highlight the need for more robust, longitudinal, and multimodal studies to further characterize the epigenetic landscape of anorexia nervosa.

### Methods

#### Population

A total of 10 girls (mean age 13.625 ± 1.79) with an active diagnosis of anorexia nervosa (AN) according to DSM-IV-TR and functional hypogonadotropic hypogonadism were enrolled at the Division of Child and Adolescent Psychiatry (eating disorder service for developmental age) of the University of Campania Luigi Vanvitelli, Naples. All patients presented with AN restrictive subtype (AN-R). Functional hypogonadotropic hypogonadism was defined as reduced levels of FSH, LH and 17-besta-estradiol or inappropriately low levels compared to clinical pubertal stage in absence of organic lesion and thyroid disfunction. Primary amenorrhea was defined as the absence of menarche five years after initial breast development; Secondary amenorrhea was defined as the cessation of previously regular menses for three consecutive months or previously irregular menses for six months.

All the patients were not under medications when DNA methylation analysis was performed, except for one patient who had started aripiprazole six months earlier. Eleven age-comparable (mean age 13.49 ± 1.48), pubertal, drug free control females without history of psychiatric or neurological disorders were also recruited. Pubertal controls were selected among patients who were referred to Pediatric Endocrinology Units of the University of Campania “Luigi Vanvitelli”, Naples for auxological or endocrinological evaluation and were finally considered healthy. Inclusion criteria for healthy controls were: BMI between 10-85th according to WHO growth chart 2006, pubertal stage, no history of menses irregularity or chronical drug assumption, and cycles irregularity if menarche already occurred. Inclusion criteria also required absence of any current or past diagnosis of eating disorders, ensuring comparability with age-matched participants diagnosed with anorexia nervosa.

Exclusion criteria for both AN and controls were:

- haematological or hepatic disorders,
- cancer.
- recent infections or surgery.

Alcohol abuse was denied by all recruited participants.

Following ethics committee approval of the study, parents have written informed consent, as well as all recruited subjects gave informed written consent to participate, before undergoing the study procedures.

### Clinical parameters

Weight, height and BMI standard deviations (SDs) were calculated using WHO growth chart 2006 for sex and age [58]. Clinical examinations were performed in all patients and controls to assess pubertal stage according to Tanner classification.

### Psychological assessment methodology

The psychological evaluation of the entire cohort of AN patients was conducted through a structured protocol comprising individual and joint clinical interviews (mother–child), family interviews, and the administration of standardized psychometric instruments. The assessment included:

- **Projective tests**: Children Apperception Test (CAT), Thematic Apperception Test (TAT), and paper-pencil drawing tasks.
- **Cognitive measures**: Wechsler Intelligence Scale for Children – Fourth Edition (WISC-IV) or Wechsler Adult Intelligence Scale (WAIS), depending on age.
- **Adaptive functioning**: Vineland Adaptive Behaviour Scales, completed by the parent.

To further investigate the presence of comorbidities and symptom profiles beyond the scope of eating disorders, a battery of self-report and caregiver-report questionnaires was administered to the minors and one caregiver. These included:

- Italian version of the Child Behaviour Checklist (CBCL) for children and adolescents aged 6 to 18 years has been administered to all AN subjects to assess behavioral and emotional problems [59].
- Children’s Eating Attitudes Test (ChEAT-26) or Eating Attitudes Test (EAT-26), a standardized self-report measure of symptoms and concerns characteristic of eating disorders [60].
- Body Uneasiness Test (BUT).
- Screen for Child Anxiety Related Emotional Disorders (SCARED).
- Cognitive Emotion Regulation Questionnaire – Youth and Adult versions (CRI-YOUTH/CRI-ADULT).
- Emotional Quotient Inventory: Youth Version (EQ-i: YV).
- The Child depression Inventory (CDI) test for in children and adolescents between the ages of 7 and 17 for comprehensive multi-rater assessment of depressive symptoms was performed to measure the cognitive, affective, and behavioral signs of depression [61]. The diagnosis of major depression was excluded based on the criteria established by the DSM-IV [15].

To assess the internal consistency of selected psychometric instruments, Cronbach’s alpha coefficients were calculated for the main measurements. Scales with a Cronbach’s alpha value equal to or greater than 0.70 were considered reliable.

### Laboratory analyses

All blood samples were drawn between 8:00 and 8:30 a.m. from an antecubital vein, clotted, centrifuged, and serum was stored at − 20 °C until analyses were performed. FSH and LH concentrations were determined by immunochemiluminometric assay (ICMA) with inter-assay coefficient of variation less than 5%. Serum 17-β-estradiol was measured by using competitive chemiluminescent immunoassay using coated magnetic particles with inter-assay coefficient of variation less than 5%.

### Samples Preparation and genome-wide DNA methylation analysis

Genomic DNA was extracted from peripheral blood leukocytes by using Wizard^®^ Genomic DNA Purification Kit (Promega) and following standard procedures. DNA quality and quantity were assessed by NanoDrop (Thermo Fisher Scientific) and electrophoresis on 1% agarose gel. Microarray analyses were performed by Genomix4life S.R.L. (Baronissi, Salerno, Italy). DNA concentration in each sample was assayed with a Qubit fluorometer. Bisulfite converted DNA (250 ng) was used for analysis of whole-genome methylation using the MethylationEPIC BeadChip (Illumina, San Diego, CA, USA). In brief, bisulfite converted DNA, using EZ DNA Methylation Kit (Zymo Research, Irvine, CA, USA) was whole-genome amplified for 20 h followed by end-point fragmentation. Fragmented DNA was precipitated, denatured and hybridized to the BeadChips for 20 h at 48 °C. The BeadChips were washed, and the hybridized primers were extended and labeled before scanning the BeadChips using the Illumina iScan system. Data analysis was performed as described in Casarotto et al. [62]. CpGs beta values were obtained using ChAMP on R (v. 4.2.0). Values of blood cell composition were obtained from the patients, and missing values were imputed with a random forest model. Singular Value Decomposition was not able to detect the blood cell composition as factor that account for a substantial fraction of variation, for this reason no corrections were performed. Only CpGs with a detection p-value < 0.01 have been considered for further analysis. CpGs were considered differentially methylated if the beta difference between the groups (Δβ) was more than 10% (|Δβ| > 0.1) and FDR values < 0.05. Further analyses were performed on the CpGs located within promoter regions, which are defined as CpGs annotated in the TSS1500, TSS200, 1 Exone, and 5’UTR. DMR analysis was performed with ChAMP using the BumpHunter model with default parameters. DMR with FDR values > 0.05 and |Δβ| < 0.05 were excluded. The canonical pathways were generated through the use of QIAGEN IPA (QIAGEN Inc., https://digitalinsights.qiagen.com/IPA) and only the ones with -log(p-value) > 1.3 were considered significant. List of regions with high affinity EREs in the human genome were downloaded from the “MOUSE AND HUMAN ERE DATABASES” (http://mapageweb.umontreal.ca/maders/eredatabase) and intersected with the genomic coordinates of the Differentially Methylated Positions (DMPs). Hypergeometric test with p-value < 0.05 was used to assess the enrichment of ERE in our DMPs. Transcription factors were found using “epigenetic Landscape In Silico deletion Analysis” (LISA - http://lisa.cistrome.org/) with default parameters. All the statistics, pie charts, and heatmaps were obtained using R.

## Supplementary Information

Below is the link to the electronic supplementary material.

Supplementary Material 1

Supplementary Material 2

Supplementary Material 3

## Data Availability

Raw methylation data and the normalized beta-values are available on ArrayExpress (E-MTAB-14823).

## Abbreviations

ALDH4A1: Aldehyde Dehydrogenase 4 Family Member A1
AMPK: AMP–Activated Protein Kinase
AN: Anorexia Nervosa
ARNTL: Aryl Hydrocarbon Receptor Nuclear Translocator Like
BAT: Brown Adipose Tissue
BMD: Bone Mineral Density
BMI: Body Mass Index
BMR: Basal Metabolic Rate
CAMKK2: Calcium/Calmodulin Dependent Protein Kinase Kinase 2
CBCL: Child Behavior Checklist
CDH7: Cadherin 7
CDI: Child Depression Inventory
CLU: Clusterin
CpG: Cytosine–phosphate–Guanine
CTBP2: C–Terminal Binding Protein 2
DMP: Differentially Methylated Position
DMR: Differentially Methylated Region
DNMT1: DNA Methyltransferase 1
DNMT3B: DNA Methyltransferase 3 Beta
DSE: Dermatan Sulfate Epimerase
DLK1: Delta Like Non–Canonical Notch Ligand 1
EAT: 26–Eating Attitudes Test
EIF4G1: Eukaryotic Translation Initiation Factor 4 Gamma 1
ERE: Estrogen Responsive Element
ESR1: Estrogen Receptor 1
EXT2: Exostosin Glycosyltransferase 2
FDR: False Discovery Rate
FHL2: Four And A Half LIM Domains 2
FSH: Follicle Stimulating Hormone
GAB2: GRB2 Associated Binding Protein 2
GHR: Growth Hormone Receptor
GHRL: Ghrelin And Obestatin Prepropeptide
GnRH: Gonadotropin–Releasing Hormone
GPCR: G Protein–Coupled Receptor
GWAS: Genome–Wide Association Study
HHLA2: HERV–H LTR–Associating 2
ICMA: Immunochemiluminometric Assay
IGF2R: Insulin Like Growth Factor 2 Receptor
IPA: Ingenuity Pathway Analysis
IRAK3: Interleukin 1 Receptor Associated Kinase 3
LEPR: Leptin Receptor
LH: Luteinizing Hormone
LTA: Lymphotoxin Alpha
LRP5: LDL Receptor Related Protein 5
MKL2: Megakaryoblastic Leukemia Translocation 2
MKRN3: Makorin Ring Finger Protein 3
NFAT5: Nuclear Factor Of Activated T–Cells 5
NOD1: Nucleotide Binding Oligomerization Domain Containing 1
NR1H3: Nuclear Receptor Subfamily 1 Group H Member 3
PR: Progesterone Receptor
PRKAG2: Protein Kinase AMP–Activated Non–Catalytic Subunit Gamma 2
PTPRD: Protein Tyrosine Phosphatase Receptor Type D
RMI1: RecQ Mediated Genome Instability 1
RORA: RAR Related Orphan Receptor A
RPTOR: Regulatory Associated Protein Of MTOR Complex 1
SFMBT1: Scm Like With Four MBT Domains 1
SORBS1: Sorbin And SH3 Domain Containing 1
TSS: Transcription Start Site
TNXB: Tenascin XB
UTR: Untranslated Region
WWP2: WW Domain Containing E3 Ubiquitin Protein Ligase 2
ZNF30: Zinc Finger Protein 30
ZNF148: Zinc Finger Protein 148
ZNF331: Zinc Finger Protein 331
ZNF365: Zinc Finger Protein 365
ZNF445: Zinc Finger Protein 445
ZNF574: Zinc Finger Protein 574
ZNF664: Zinc Finger Protein 664
ZNF860: Zinc Finger Protein 860
ZFAND2B: Zinc Finger AN1–Type Containing 2B
ZFAT: Zinc Finger And AT–Hook Domain Containing
ZFP57: Zinc Finger Protein 57
ZBTB43: Zinc Finger And BTB Domain Containing 43
ZMIZ2: Zinc Finger MIZ–Type Containing 2
